# Development of digital breast tomosynthesis and diffuse optical tomography fusion imaging for breast cancer detection

**DOI:** 10.1038/s41598-020-70103-0

**Published:** 2020-08-04

**Authors:** Eun Young Chae, Hak Hee Kim, Sohail Sabir, Yejin Kim, Hyeongseok Kim, Sungho Yoon, Jong Chul Ye, Seungryong Cho, Duchang Heo, Kee Hyun Kim, Young Min Bae, Young-Wook Choi

**Affiliations:** 1 Department of Radiology, Research Institute of Radiology University of Ulsan College of Medicine, Asan Medical Center, 88, Olympic-ro 43-Gil, Songpa-Gu, Seoul, 05505 South Korea; 2 Department of Nuclear and Quantum Engineering, Korea Advanced Institute of Science and Technology (KAIST), 291, Daehak-ro, Yuseong-gu, Daejeon, 34141 South Korea; 3 Department of Bio and Brain Engineering, Korea Advanced Institute of Science and Technology (KAIST), 291, Daehak-ro, Yuseong-gu, Daejeon, 34141 South Korea; 4 KAIST Institutes for ITC, HST, and AI, Korea Advanced Institute of Science and Technology (KAIST), 291, Daehak-ro, Yuseong-gu, Daejeon, 34141 South Korea; 5 Korea Electrotechnology Research Institute (KERI), 111, Hanggaul-ro, Sangnok-gu, Ansan, 15588 South Korea

**Keywords:** Cancer, Oncology

## Abstract

Diffuse optical tomography (DOT) non-invasively measures the functional characteristics of breast lesions using near infrared light to probe tissue optical properties. This study aimed to evaluate a new digital breast tomosynthesis (DBT)/DOT fusion imaging technique and obtain preliminary data for breast cancer detection. Twenty-eight women were prospectively enrolled and underwent both DBT and DOT examinations. DBT/DOT fusion imaging was created after acquisition of both examinations. Two breast radiologists analyzed DBT and DOT images independently, and then finally evaluated the fusion images. The diagnostic performance of each reading session was compared and interobserver agreement was assessed. The technical success rate was 96.4%, with one failure due to an error during DOT data storage. Among the 27 women finally included in the analysis, 13 had breast cancer. The areas under the receiver operating characteristic curve (AUCs) for DBT were 0.783 and 0.854 for readers 1 and 2, respectively. DOT showed comparable diagnostic performance to DBT for both readers. The AUCs were significantly improved (P = 0.004) when the DBT/DOT fusion images were used. Interobserver agreements were highest for the DBT/DOT fusion images. In conclusion, this study suggests that DBT/DOT fusion imaging technique appears to be a promising tool for breast cancer diagnosis.

## Introduction

Although conventional mammography remains the primary screening tool for breast cancer and has been shown to reduce breast cancer mortality in randomized trials^1,2^, it is subject to the obvious limitations of low sensitivity and specificity in women with dense breast tissues^3–5^. Digital breast tomosynthesis (DBT), which was approved by the FDA in 2011, is rapidly emerging as the new standard of care for breast imaging. Results from retrospective studies^6–8^ and prospective trials^9–11^ have demonstrated that by separating overlying breast tissue, DBT increases cancer detection and decreases recall rate. However, it also allows only morphological assessment of breasts, and the improvement in outcomes with DBT is mostly related to women with heterogeneously dense breasts, with the improvement achievable with DBT being limited in extremely dense breasts^12^.

Alternative imaging modalities providing functional assessment of tissue to anatomical X-ray imaging could potentially lead to improvements in breast cancer detection. Several additional techniques including magnetic resonance imaging (MRI) and contrast-enhanced spectral mammography have been introduced to identify abnormalities on the basis of angiogenesis; however, most of these require the intravenous injection of contrast materials for imaging. Diffuse optical tomography (DOT) is a non-invasive imaging modality that uses near infrared light to probe the optical properties of tissue, and it can be used to measure the functional characteristics of breast lesions. Near infrared light projected into tissue is scattered and absorbed by endogenous chromophores such as oxygenated hemoglobin, deoxygenated hemoglobin, water, and lipids. Healthy and diseased tissues can be differentiated on DOT according to their different absorption or scattering coefficients. Unfortunately, the low spatial resolution of DOT resulting from its high sensitivity to noise is one of the major challenges limiting its clinical application^13,14^. To this end, there have been efforts to improve the quality of reconstructed DOT images by introducing hybrid or image-guided DOT systems for breast cancer imaging^15–24^.

In this study, we developed DBT and DOT fusion imaging for breast cancer detection. We hypothesized that DBT/DOT fusion imaging would be useful for breast cancer diagnosis, with the technique combining both the morphological features provided by DBT and the functional information provided by DOT. The purpose of this study was to evaluate a new DBT/DOT fusion imaging technique and obtain preliminary data on its suitability for breast cancer detection, comparing it with DBT and DOT alone.

## Methods

### Study design and participants

This prospective study was approved by institutional review board of Asan Medical Center and written informed consent was obtained from all participating patients. All experiments were carried out in accordance with relevant guidelines and regulations. Patient inclusion was based on the following criteria: (1) patients were at least 40 years-of-age; (2) patients had histopathologic results or clinical follow-up of at least 2 years; and (3) patients agreed to participate voluntarily. Patients with a history of a previous breast operation or with foreign materials such as implants were not eligible. Between August 2018 and December 2018, 28 women were enrolled in this study and underwent both DBT and DOT examinations. The data from one subject were excluded from the image analysis because of an error in the data storage process. Finally, a total of 27 women (age range 40–69 years; mean age, 52.6 years) formed our study population. The reference standards were from surgery (n = 11), clinical follow-up (n = 10), or core needle biopsy (n = 6).

### DBT and DOT examinations

1. DBT system.
  DBT examinations were performed with a commercially available system (SenoClaire, GE Healthcare, Waukesha, WI). The bilateral two views, craniocaudal (CC) and mediolateral oblique (MLO), were obtained. The system acquired nine projection images along a − 12.5° to + 12.5° arc using a step-and-shoot technique. These low-dose images were reconstructed into thin slices of the breast using iterative reconstruction (ASiR, Adaptive Statistical Iterative Reconstruction, GE Healthcare, Waukesha, WI). The mean compressed breast thickness was 51.2 ± 10.6 mm (range 20–74 mm).
2. Multi-channel DOT system.
  The multi-channel DOT system was developed at the Korea Electrotechnology Research Institute^25^. The schematics of the multi-channel DOT system are shown in Fig. 1. The system consists of a light source, optical detector, optical probe, data acquisition board, and controller. The frequency domain DOT system is adapted with three optical fiber pigtailed laser diodes (wavelengths; 785 nm, 808 nm, and 850 nm) modulated at a 70 MHz radiofrequency signal. The amplitude should be adjusted for object conditions because detection power should be in the detection dynamic range. The beam diameter is 1.4 mm after collimators. Two optical MEMS (micro-electro-mechanical system) switches are used to deliver light from 3 laser diodes to 64 specific positions in the source paddle. During the optical switching, single-tone modulation light photons reach 40 detection fiber ends in the detection paddle and are detected simultaneously by 40 avalanche photodiodes (APD). The APD has active area with a diameter of 3 mm and 80 MHz 3 dB bandwidth. The DOT system uses in-phase (I) and quadrature (Q) demodulators to obtain the amplitude and phase of the signal in the signal processing card. The 40 IQ signal pairs are simultaneously acquired using data acquisition boards. This multi-channel DOT system obtains craniocaudal views of both breasts.

**Figure 1:**
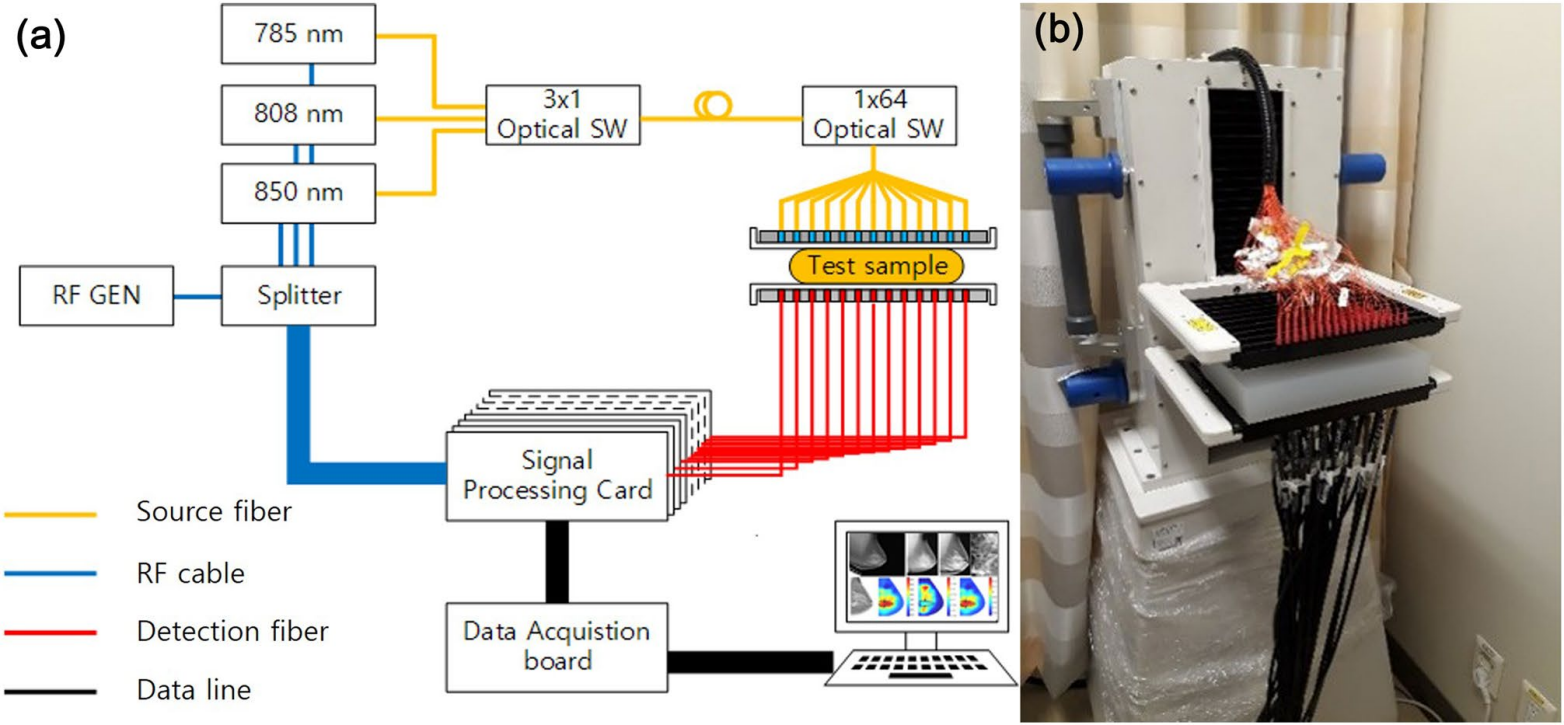
Multi-channel diffuse optical tomography (DOT) system. Schematic view (**a**) and photograph (**b**) of the DOT system. The light source provides three laser wavelengths (785 nm, 808 nm, and 850 nm) modulated at a 70 MHz radiofrequency signal. Two optical switches are used to deliver light to 64 positions in the source paddle. Single-tone modulation light photons reach 40 detection fiber ends and are detected by 40 avalanche photodiodes installed in the home-made signal processing card.
3. Data processing and reconstruction of DBT/DOT fusion images.
  The core structure of the DOT iterative image reconstruction^26^ was inspired by NIRFAST software^27^. The preprocessing, calibration, bulk optical property estimation, and maximum intensity projection (MIP) processes were additively implemented in accordance with the developed hardware system.
  1. Preprocessing and calibration.
    The raw data were rearranged into amplitude and phase by taking the complex form in the first place. The amplitude term plays a central role in selecting useful data for the image reconstruction. As the detector array and source array are mounted on panels in parallel geometry, air gaps between the breast and each panel are unavoidable in the peripheral regions of the breast. Such air gaps near the peripheral region of the breast introduce reflections or surface scatter of the optical beam, rather than transmission, and the detected signal may consequently be severely distorted. For example, the beam from source 2 would successfully propagate through the breast tissues without air distortion, while the beam from source 7 would be largely reflected or scattered at the boundary between the air and the skin because of the existing air-gap (Fig. 2). Likewise, the signals detected at peripheral regions with air gaps, such as signals detected by the detectors from (e) to (i), would include unwanted tissue-irrelevant scattered photons (Fig. 2). Therefore, it would be better to discard these signals with air gap contamination, unless a sophisticated modeling technique, which is beyond the scope of this work, were to be included in the image reconstruction. The selected data went through a calibration process^28^. The DOT system uses homodyne detection technology at a 70 MHz single tone. Thus there inherently are the physical variations in the power of light sources, the sensitivity of detectors, phase of RF stripline and cable, gain, attenuation, and loss of RF components like RF AMP, connector, attenuator, and so on. These variations make variations in IQ signals in source-detector pairs despite the same optical path in an optically homogenous calibration phantom with known geometry and optical properties including scattering and absorption coefficients. The purpose of the calibration was to compensate for the variations in IQ signal in source-detector pairs. In this study, we measured IQ signals of all channels using the calibration phantom, and then we compensated for offsets of IQ signals from the mean of IQ signals. In this manner, calibration factors were prepared for each source–detector pairing. The correction factor was determined for each detection channel as following:

**Figure 2:**
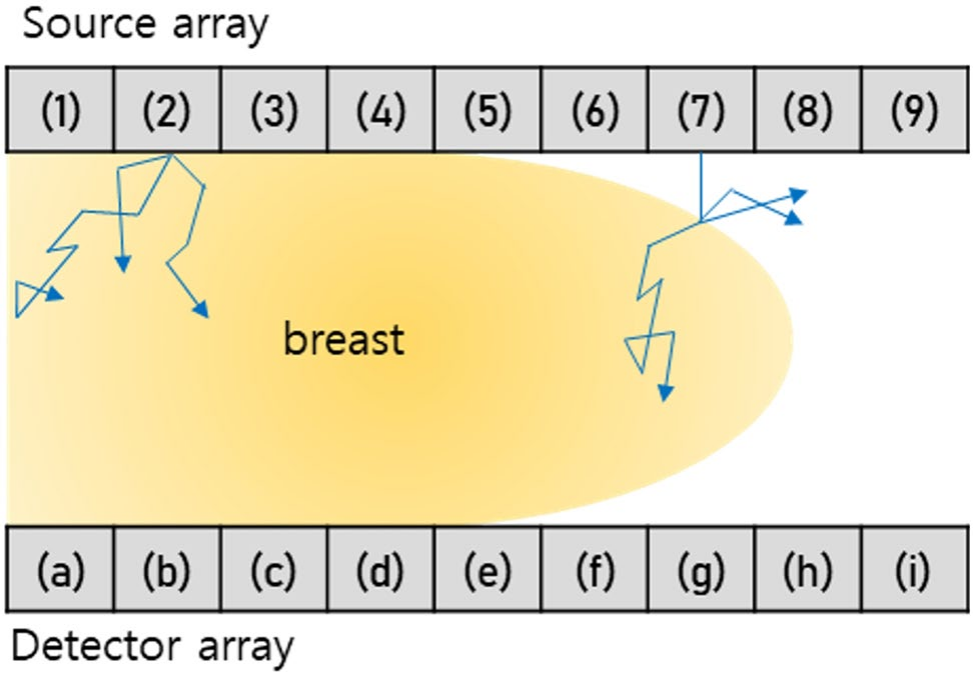
A schematic illustration of the DOT scanning system.
    $${\mathrm{CF}}_{\mathrm{amplitude}}=\frac{\mathrm{amplitude}}{mean\left(\mathrm{amplitude}\right)} \mathrm{and} $$$${\mathrm{CF}}_{\mathrm{phase}}=\frac{\mathrm{phase}}{mean\left(\mathrm{phase}\right)}$$.
    Across the 40 detector channels, we observed an overall uniform response. In the clinical study, we have performed this calibration before scanning each patient since the DOT system was off for a substantial time between patient scans.
    We performed the clinical test after enough warming up of the DOT system because the properties of optical and electrical components depend on the temperature. Notably, the laser diodes should be operated in constant temperature conditions under TEC (thermoelectric cooler). We measured the calibration offset data for each clinical test because the sensitivities of APDs depend on the temperature despite enough warming-up time.
  2. Bulk optical property estimation.
    Using the sorted and calibrated data described above, the bulk optical property was estimated and used as an initial guess for the iterative reconstruction. We noticed that the reconstruction results are sensitive to the accuracy of the initial bulk optical property estimation, and thus a careful estimation of the initial bulk optical property is required ahead of the reconstruction step. The bulk optical properties were calculated based on the slope between the processed data and the source-detector distance.
    1 $$\underset{{\mu }_{a}, { \mu }_{s}}{\mathrm{argmin}} {\left[\frac{\mathrm{d}\left(\mathrm{ln}\left(\rho {\varphi }^{meas}\right)\right)}{\mathrm{d\rho }}-\frac{\mathrm{d}\left(\mathrm{ln}\left(\rho {\varphi }^{cal}\right)\right)}{\mathrm{d\rho }}\right]}^{2}+{\left[\frac{\mathrm{d}{\theta }^{meas}}{\mathrm{d\rho }}-\frac{\mathrm{d}{\theta }^{cal}}{\mathrm{d\rho }}\right]}^{2}.$$
    where $\varphi$ refers to the amplitude, $\uprho$ the source-to-detector distance, and $\theta$ the phase. This minimization can be readily solved using the Newton-Rapson method^29^.
  3. Reconstruction and maximum intensity projection (Fig. 3).

**Figure 3:**
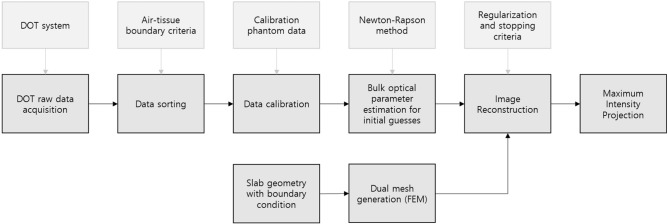
Schematic diagram of DOT image reconstruction process.
    The reconstruction of the DOT image in our developed prototype involves solving the diffusion equation:
    2 $$-\nabla \bullet\upkappa \left(\mathrm{r}\right)\nabla\Phi \left(\mathrm{r},\upomega \right)+\left({\mu }_{a}\left(r\right)+\frac{i\omega }{{c}_{m}\left(r\right)}\right)\Phi \left(\mathrm{r},\upomega \right)={q}_{o}\left(\mathrm{r},\upomega \right), $$
    where ${\mu }_{a}$ is the absorption coefficient, $\upkappa \left(\mathrm{r}\right)=\frac{1}{3({\mu }_{a}+{\mu }_{s}\mathrm{^{\prime}})}$ is the diffusion coefficient at position $\mathrm{r}$ with the reduced scattering coefficient ${\mu }_{s}\mathrm{^{\prime}}$, and $\Phi \left(\mathrm{r},\upomega \right)$ is the photon fluence at position $\mathrm{r}$ with a modulation frequency $\upomega$. ${c}_{m}(r)$ is the speed of light in the medium at point $r$, and ${q}_{o}(\mathrm{r},\upomega )$ represents an isotropic source term. We applied the Robin boundary condition for the air-tissue boundary representation, which assumes that the fluence at the air-tissue boundary exits to the air but does not return.

The photon fluence can be rewritten as $\Phi \left(\mathrm{r},\upomega \right)={\Phi }_{0}\left(\mathrm{r},\upomega \right)+{\Phi }_{\mathrm{sca}}\left(\mathrm{r},\upomega \right)$ or $\Phi \left(\mathrm{r},\upomega \right)={\Phi }_{0}\left(\mathrm{r},\upomega \right){e}^{{\Phi }_{\mathrm{sca}}^{R}\left(\mathrm{r},\upomega \right)}$ according to the Born and Rytov approximation, where ${\Phi }_{0}\left(\mathrm{r},\upomega \right)$ is the fluence in the homogenous background and ${\Phi }_{\mathrm{sca}}\left(\mathrm{r},\upomega \right)$ or ${\mathrm{e}}^{{\Phi }_{\mathrm{sca}}^{R}\left(\mathrm{r},\upomega \right)}$ is the correction term for heterogeneities^13,30^. These forms can be used to establish an inverse problem for DOT reconstruction.

We utilized the Levenberg–Marquardt (LM) optimization algorithm, which is the most used algorithm for reconstructing DOT images iteratively^27,31^. The algorithm requires the experimental data, a bulk optical property estimation for initial guesses, regularization, and stopping criteria for terminating the iterations. The regularization parameter affects the smoothness of the reconstructed images. In this study, the iteration stopped when the updates for the whole images were considered marginal. The image reconstruction, which involves forward and inverse models, is carried out on a dual-mesh scheme in the finite element method (FEM) structure. The FEM is normally used to solve partial differential equations in various engineering fields, including biomedical optics^32,33^. The distance between two nodes was typically 2–5 mm, while a finer mesh was used to solve the forward problem and a coarser mesh was used for the inverse problem. To compare the DOT images to the mammographic images, the maximum value along the depth (z-)direction was projected into a 2-dimensional image domain, which is referred to as a MIP image.

### Image analysis

Two board-certified breast radiologists with 9 and 22 years of breast imaging experience participated in the image analysis. The readers were fellowship-trained, dedicated breast radiologists and had more than 7 years of experience with DBT. They also had experience with DOT using biomimetic phantoms and animals, and had participated in previous study involving DOT technology^19^. Each reader independently completed three separate sessions: (1) reconstructed DBT alone, (2) reconstructed DOT alone, and (3) DBT/DOT fusion images. The readers were blinded to the readings of the other radiologist, as well as the clinical and histopathologic information. Patient records and information were anonymized and de-identified prior to analysis.

In each reading session, the readers were asked to detect the presence or absence of abnormalities that suggested breast cancer. In the first reading session (i.e., DBT alone), the readers evaluated DBT images in accordance with the Breast Imaging Reporting and Data System (BI-RADS) lexicon^34^. If a lesion of BI-RADS category 4 or higher was detected, the test was considered “positive”. In the second reading session (i.e., DOT alone), the readers evaluated both breasts simultaneously and assessed whether there was unique activity different from that in the surrounding background parenchyma. Each DOT image reflects the optical properties of the tissue according to the absorption coefficients of the corresponding areas, which are represented as a color map. In the third reading session, the readers evaluated the DBT/DOT fusion images using the DBT as an anatomical reference. In all reading sessions, the interpretation results were dichotomized into “positive” or “negative”. The readers were not allowed to modify the results evaluated in the other reading sessions.

The lesion types were also determined according to the DBT, being classified into mass or calcification only, mass with calcification, an architectural distortion, or asymmetry. Overall mammographic breast density was recorded for each patient according to the four categories of breast composition described by the American College of Radiology (ACR) BI-RADS^34^.

### Statistical analysis

All statistical analyses were performed using SPSS software (version 23.0, Statistical Package for the Social Sciences, Chicago, IL). A P value of < 0.05 was considered statistically significant. The diagnostic performance of the parameters, including sensitivity, specificity, positive predictive value (PPV), and negative predictive value (NPV), was analyzed for each reading session. The diagnostic ability for breast cancer on each imaging modality was investigated using the area under the receiver operating characteristic curve (AUC). The diagnostic performance of each reading session was compared using McNemar’s or Fisher’s exact test, or a logistic model with generalized estimating equations, and AUCs were compared using the DeLong test. Reader agreement in the image analyses was determined using the Fleiss ĸ statistic, with ĸ = 1.0 denoting perfect agreement; 0.81–0.99, almost perfect agreement; 0.61–0.80, substantial agreement; 0.41–0.60, moderate agreement; 0.21–0.40, fair agreement; and < 0.20, slight agreement^35^.

## Results

### Patients and lesion characteristics

**Figure 4:**
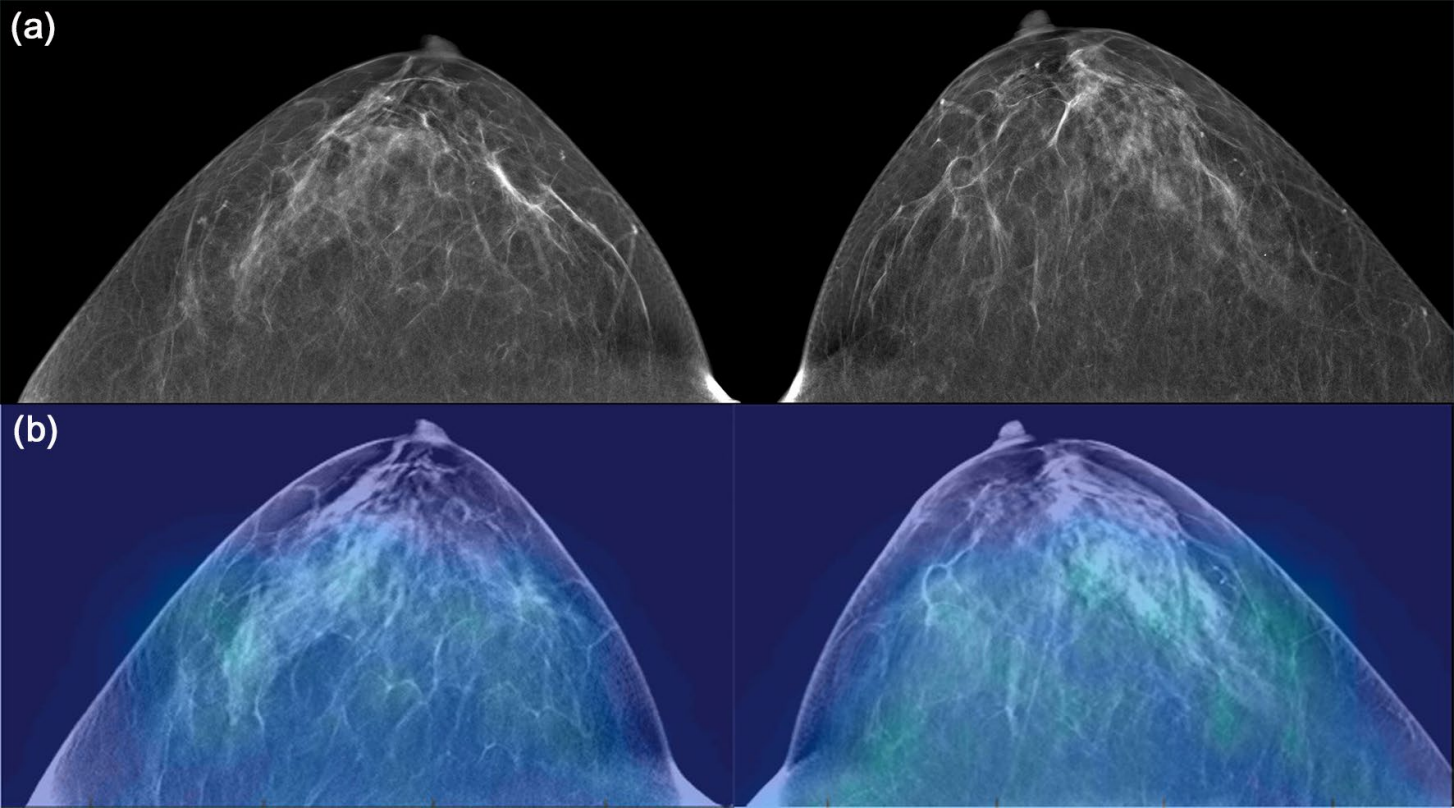
A 41-year-old woman with normal fatty breast. Digital breast tomosynthesis (DBT) images (**a**) show scattered areas of fibroglandular density without abnormal mass or significant calcification. Reconstructed DBT/DOT fusion images (**b**) show homogeneous symmetric activity in both breasts. *The images in this figure and Figs. 5, 6, 7 and 8 were generated using the Photoshop software (version 6.0; Adobe Systems, Palo Alto, CA).

**Figure 5:**
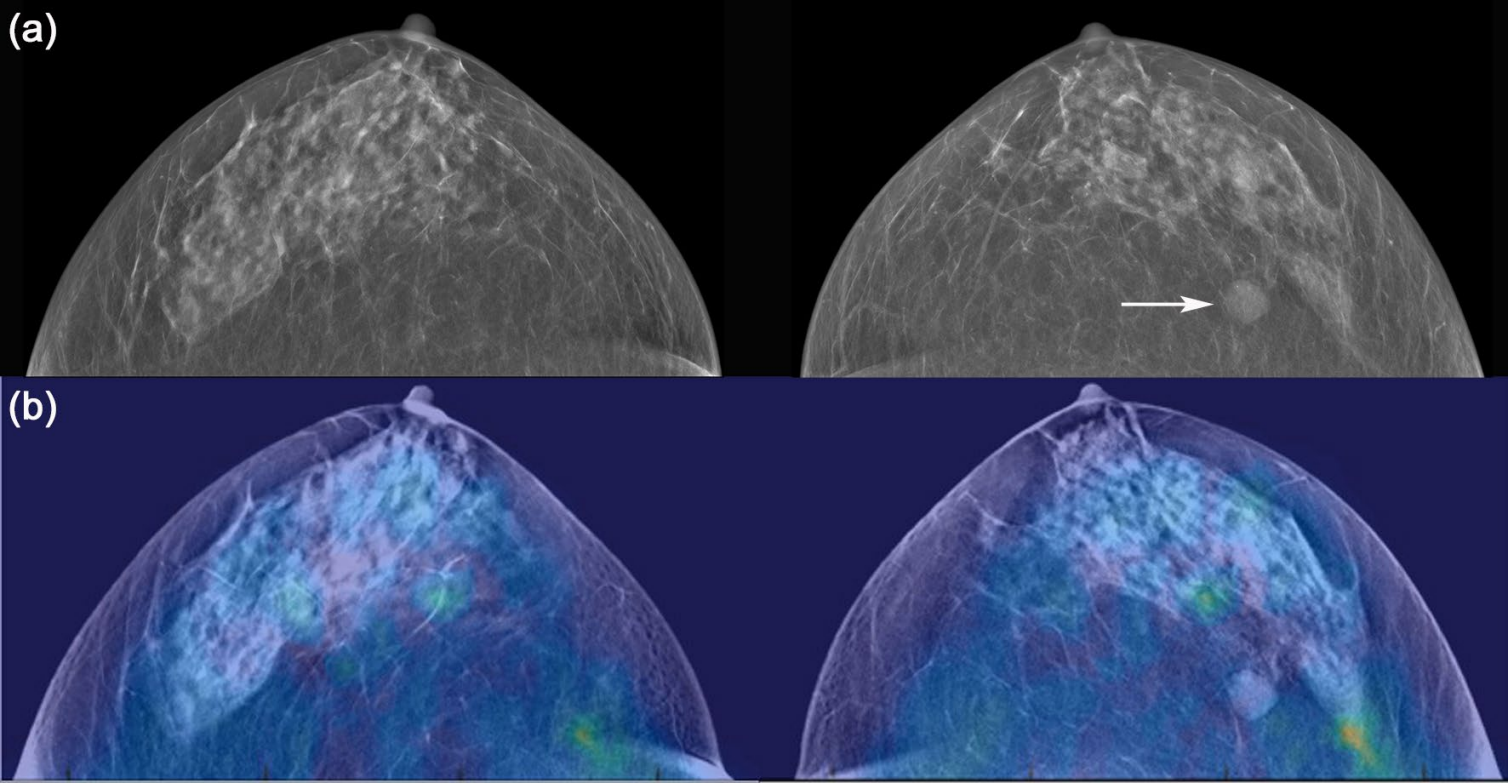
A 61-year-old woman with a benign mass in the left breast. DBT images (**a**) show a circumscribed isodense mass (arrow) in the outer deep portion of the left breast, suggesting a probably benign lesion. Reconstructed DBT/DOT fusion images (**b**) show no focal increased activity in either breast. The mass was unchanged for 10-year follow-up.

**Figure 6:**
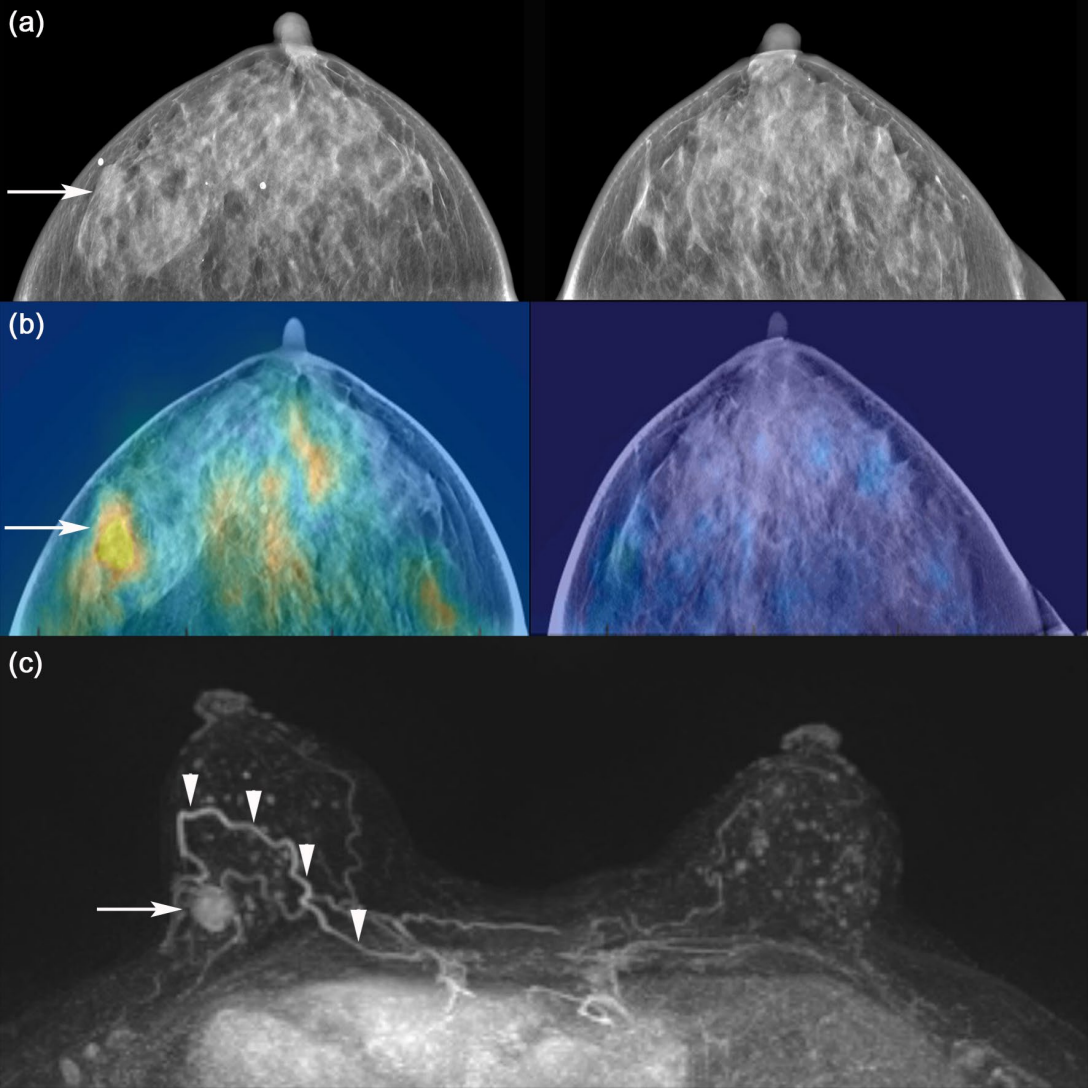
A 52-year-old woman with an invasive ductal carcinoma in the right breast. DBT images (**a**) show an irregular shaped mass (arrow) in the right outer breast. Reconstructed DBT/DOT fusion images (**b**) show asymmetrically increased activity (arrow) in the corresponding area of the right breast. On breast magnetic resonance imaging (MRI) (**c**), an enhancing mass (arrow) with heterogeneous enhancement is seen on maximum intensity projection (MIP) image. A dominant feeding vessel (arrowheads) with its origin in the internal mammary artery is also clearly visible. Surgery confirmed invasive ductal carcinoma.

**Figure 7:**
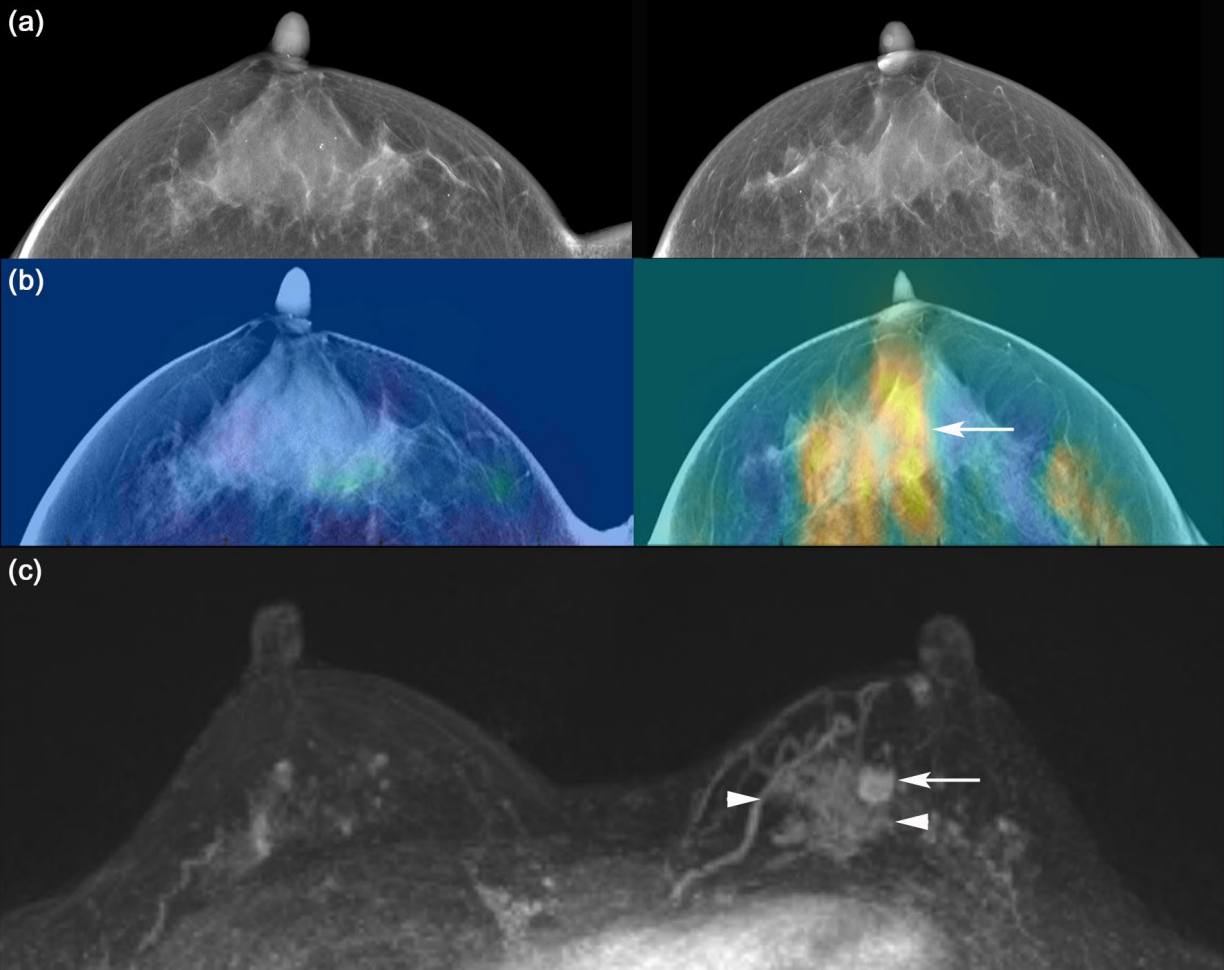
A 40-year-old woman with an invasive ductal carcinoma and accompanying ductal carcinoma in situ in the left breast. DBT images (**a**) show extremely dense breasts without a definite abnormal focal lesion. Reconstructed DBT/DOT fusion images (**b**) show an increased absorption coefficient in the left breast (arrow) compared with the contralateral breast. Breast MRI (**c**) shows a 1-cm enhancing mass (arrow) associated with indeterminate nonmass enhancement (arrowheads) in the left inner breast. Surgery confirmed invasive ductal carcinoma and accompanying ductal carcinoma in situ.

DBT and DOT examinations were successfully completed in 27 of 28 patients, with an error occurring during the DOT data storage process for one patient; therefore, the technical success rate was 96.4%. Among the 27 women finally included in the analysis, 13 (48.1%) had breast cancers, eight had benign breast disease, and six had normal findings (Figs. 4, 5, 6, 7). The malignant tumors were predominantly invasive ductal carcinomas (10/13; 76.9%), with the other types being two ductal carcinomas in situ and one microinvasive ductal carcinoma. On DBT, 16 of 27 (59.3%) women had heterogeneously dense or extremely dense breast tissue, while 11 subjects had scattered areas of fibroglandular density or almost entirely fatty breasts. Nine of the 13 breast cancer lesions appeared as a mass on DBT, three appeared as a mass with calcification, and one as a calcification only without an accompanying mass. Of the eight benign breast lesions, seven appeared as a mass and one as calcification only.

### Performance parameters of the DBT, DOT, and DBT/DOT fusion images

Table 1 presents the performance parameters of DBT, DOT, and DBT/DOT fusion images for each reader, and comparisons between the image types. The sensitivity, specificity, PPV, and NPV of DBT were 92.3%, 64.3%, 70.6%, and 90.0%, respectively, for reader 1, and 92.3%, 78.6%, 80.0%, and 91.7% for reader 2. The sensitivity, specificity, PPV, and NPV of DOT were 61.5%, 85.7%, 80.0%, and 70.6%, respectively, for reader 1, and 61.5%, 78.6%, 72.7%, and 68.8% for reader 2. The sensitivity, specificity, PPV, and NPV of the DBT/DOT fusion images were 100%, 92.9%, 92.9%, and 100%, respectively, for reader 1, and 100%, 85.7%, 86.7%, and 100% for reader 2. The differences between the imaging modalities were not statistically significant, except for the specificity and PPV for reader 1.

**Table 1:** Performance parameters for DBT vs. DOT vs. DBT/DOT fusion images for each reader.

	DBT	DOT	DBT/DOT fusion image	P value		
				DBT vs. DOT	DBT vs. DBT/DOT	DOT vs. DBT/DOT
**Reader 1**						
Sensitivity	92.3 (66.7–98.6)	61.5 (35.5–82.2)	100 (77.2–100)	0.219	1.000	0.063
Specificity	64.3 (38.8–83.7)	85.7 (60.1–95.9)	92.9 (68.5–98.7)	0.176	0.043*	0.311
Positive predictive value	70.6 (46.9–86.7)	80.0 (49.0–94.3)	92.9 (68.5–98.7)	0.361	0.003*	0.117
Negative predictive value	90.0 (59.6–98.2)	70.6 (46.9–86.7)	100 (77.2–100)	0.363	0.435	0.052
**Reader 2**						
Sensitivity	92.3 (66.7–98.6)	61.5 (35.5–82.3)	100 (77.2–100)	0.219	1.000	0.063
Specificity	78.6 (52.4–92.4)	78.6 (52.4–92.4)	85.7 (60.1–95.9)	1.000	0.563	0.304
Positive predictive value	80.0 (54.8–92.9)	72.7 (43.4–90.3)	86.7 (62.1–96.3)	0.791	0.244	0.111
Negative predictive value	91.7 (64.6–98.5)	68.8 (44.4–85.8)	100 (75.8–100)	0.196	1.000	0.053

Numbers in parenthesis are 95% confidence interval.

*P values less than 0.05.

**Figure 8:**
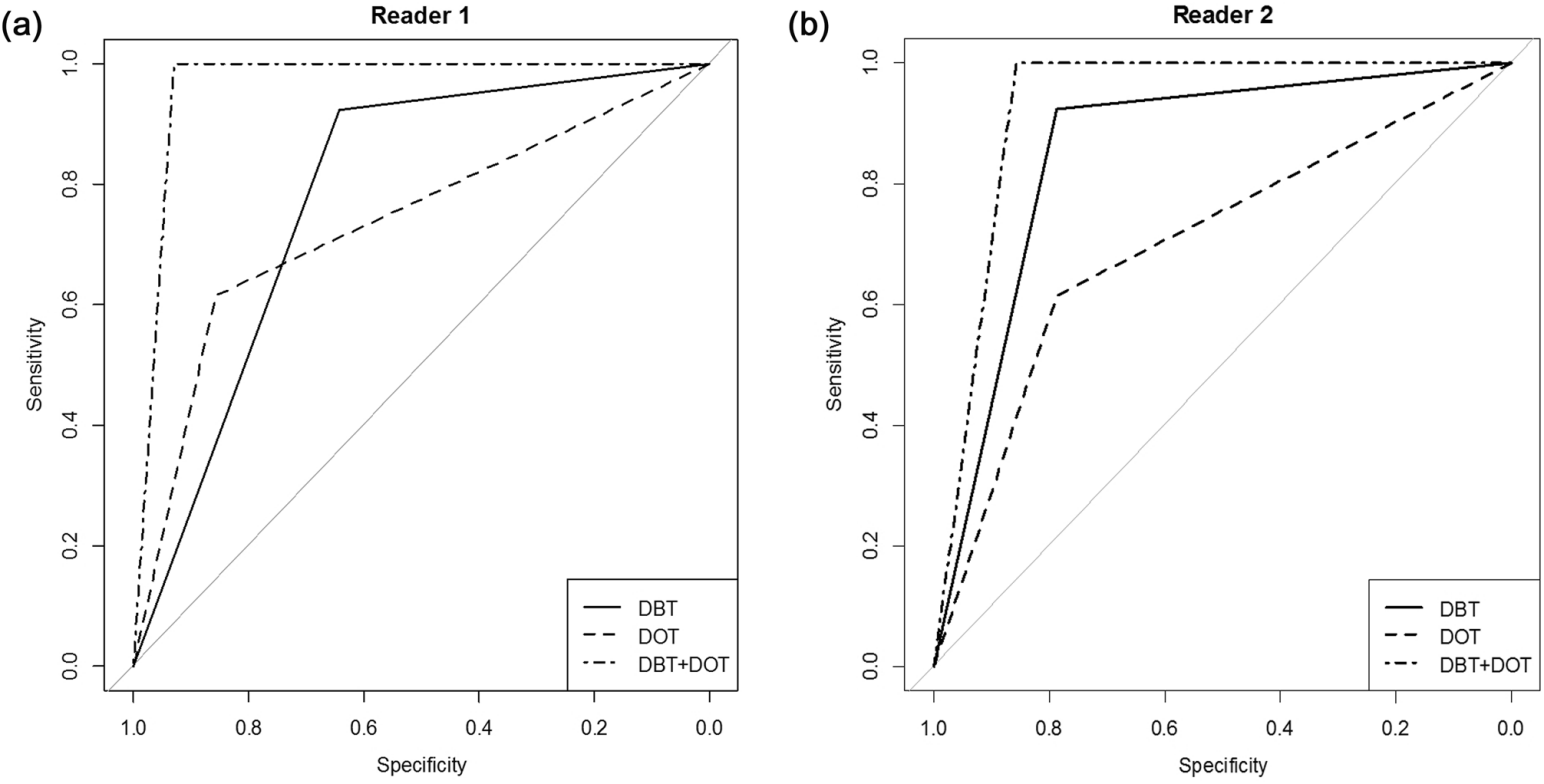
Receiver operating characteristic curves of DBT vs. DOT vs. DBT/DOT fusion images for reader 1 (**a**) and reader 2 (**b**).

Table 2 summarizes the average AUCs for the diagnosis of breast cancer for each imaging modality (Fig. 8). The AUCs for DBT were 0.783 and 0.854 for readers 1 and 2, respectively. The DOT showed comparable diagnostic performance to DBT, with AUCs of 0.736 and 0.701 for readers 1 and 2, respectively (P = 0.689 and 0.180). When the DBT/DOT fusion images were used, the AUCs showed improvement to 0.964 and 0.929 for readers 1 and 2 respectively. The DBT/DOT fusion images showed significantly higher AUCs than DOT alone (P = 0.004 for both readers), but the differences between DBT/DOT fusion images and DBT alone reached statistical significance only for reader 1 (P = 0.014), not for reader 2 (P = 0.317).

**Table 2:** Areas under the curve for distinguishing breast cancer for each imaging modality.

	DBT	DOT	DBT/DOT fusion image	P value		
				DBT vs. DOT	DBT vs. DBT/DOT	DOT vs. DBT/DOT
Reader 1	0.783 (0.632–0.933)	0.736 (0.569–0.904)	0.964 (0.894–1)	0.689	0.014*	0.004*
Reader 2	0.854 (0.720–0.989)	0.701 (0.523–0.878)	0.929 (0.833–1)	0.180	0.317	0.004*

Numbers in parenthesis are 95% confidence interval.

*P values less than 0.05.

Table 3 shows the interobserver agreements between the radiologists for the imaging analyses. The interobserver agreements for evaluating DBT and DOT alone were substantial, with a ĸ value of 0.695 (95% confidence interval (CI) 0.423–0.967) for DBT alone and 0.767 (95% CI 0.519–1.000) for DOT alone. The ĸ value increased to 0.926 (95% CI 0.783–1.000) for DBT/DOT fusion images, indicating almost perfect agreement.

**Table 3:** Interobserver agreement in the imaging analyses.

Imaging method	ĸ value	95% Confidence interval^a^
DBT alone	0.695	0.423–0.967
DOT alone	0.767	0.519–1.000
DBT/DOT fusion image	0.926	0.783–1.000

^a^Bootstrapped 95% confidence intervals.

## Discussion

We evaluated our new DBT/DOT fusion imaging system for breast cancer detection in a clinical setting with both normal breasts and breasts with lesions. The technical success rate was 96.4% (27 of 28 participants). In this study, DOT alone showed comparable diagnostic performance to DBT alone. The DBT/DOT fusion images showed significantly higher diagnostic performance than DOT for both readers.

The optical imaging technique allows discrimination of malignant tumors according to different light-tissue interactions, which are expressed as absorption and scattering coefficients. One of the most promising applications for the technique is the breast, a superficial organ where the remodeled vasculature and changes caused by malignant lesions create a contrast suitable for optical imaging^36,37^. Several exploratory studies including small samples of patients have been reported. Choe et al.^38^ reported an ROC analysis using total hemoglobin as a criterion for diagnosis. They found a sensitivity of 98% and specificity of 90% for distinguishing malignant from benign lesions. Poplack et al.^39^ achieved an AUC of 0.88 for differentiating between cancer and normal tissue, and an AUC of 0.76 for differentiating between malignant and benign lesions. In our present study, including both normal tissue and malignant and benign lesions, DOT showed AUCs for distinguishing breast cancer of 0.736 and 0.701 for the two readers. One noteworthy finding is that these results were comparable to those of DBT for both readers.

DOT imaging in general falls into an ill conditioned problem and its robust image reconstruction is in general difficult. With the fine tuning of the regularization, we paid attention to the mesh generation process to empirically acquire more robust condition for each image reconstruction via the dual-mesh scheme. The two layer (breast and chest-wall) model was not considered in this work^40^, but we conjecture that the inclusion of the two layer model may improve the accuracy of the signal recovery particularly near the chest wall. In the system hardware design, when switching three-wavelength light sources, we apply a simultaneous data acquisition technique for 40 detection channels not only to reduce the clinical test time using PXIe-6368 DAQ, but also to improve stability in data acquisition for the same switching photon. The clinical test duration is within 20 s for three-wavelength laser diode channels. In this study, our focus is rather on demonstrating clinical evidences of the utility of DBT/DOT fusion imaging in a dense breast dominant cohort, using the reader study.

The major challenge for DOT imaging is that photon scattering makes it difficult to localize tumor location and size. The DOT imaging data can be combined with other imaging modalities to improve the DOT reconstruction. Using such methods, the conventional imaging modality provides morphologic guidance in the breast, which is used in the reconstruction of the optical properties of the breast. This allows the low spatial resolution and blurring of DOT images to be overcome. Several previous studies have reported hybrid techniques with other imaging modalities, including ultrasound, computed tomography, and MRI^15,20–23,41–44^. The first use of a combined optical and X-ray system of the breast was reported at Massachusetts General Hospital^45,46^. Fang et al. found a linear correlation between total hemoglobin and fibroglandular volume fraction on DBT, and a significant difference in total hemoglobin concentration between malignant tumors and solid benign lesions^46^. Krishnaswamy et al. proposed a continuous wave near infrared spectral tomography (NIRST)-DBT system using large-area silicon photodiodes^28^. When the DBT/DOT fusion images were used in our study, the sensitivity, specificity, PPV, and NPV were 100%, 85.7–92.9%, 86.7–92.9%, and 100%. The diagnostic performances for distinguishing breast cancer were significantly improved when the DBT/DOT fusion images were used (0.964 and 0.929 for readers 1 and 2, respectively).

We found that the interobserver agreement between the radiologists was higher for the DBT/DOT fusion images (ĸ value, 0.926) than for DBT or DOT alone (0.695 and 0.767, respectively). Although objective image analysis is one of the fundamental factors for determining the significance of observed findings, the presence of marked observer variability in mammographic interpretation is well known^47^. Even with the BI-RADS lexicon, variability in mammographic interpretation can still be observed, and it may be attributed both to differences in lesion detection and lesion characterization^48^. The ability to fuse the optical imaging and tomosynthesis using our new system could lead to a lower barrier for acceptance by radiologists, allowing greater confidence in the interpretation of the optical images, similar to the situation with PET/CT^49^. By viewing the fused optical and tomosynthesis images, the radiologist can map the suspicious findings from DBT with their corresponding DOT images and use the functional information for more reliable interpretation.

There are several limitations to our study which should be considered in the interpretation of the study results. First, this study included only 27 participants, and only a few of the evaluated performance measures showed significant improvements with the DBT/DOT fusion images, despite the generally positive trends. The small sample size precluded the use of more powerful statistical methods for detecting changes. Given the limited number of patients involved in this study, the results reported here should be considered preliminary, and a trial with a larger sample size is required to further explore the favorable trends observed in this study. Second, in the present study we used a commercially available DBT system and our newly developed DOT system. We realize that a more accurate evaluation and one-to-one comparison between the DBT and DOT would be possible if the two images were acquired simultaneously under a single breast compression. Furthermore, we analyzed the DBT and DOT images using dichotomized criteria in this study. More rigorous assessments including quantitative measurement of DOT images are needed.

In this study, we evaluated our new DBT/DOT fusion imaging system in a clinical setting. The study suggests that DBT/DOT fusion imaging appears to be a promising tool in noninvasive breast cancer detection. The improved discrimination of malignant breast lesions is a motivation for further advancement of this new imaging technique. A larger trial is warranted to clarify the clinical usefulness of DBT/DOT fusion imaging.
